# Balibalosides, an Original Family of Glucosylated Sesterterpenes Produced by the Mediterranean Sponge *Oscarella balibaloi*

**DOI:** 10.3390/md11051477

**Published:** 2013-05-06

**Authors:** Coralie Audoin, Dominique Bonhomme, Julijana Ivanisevic, Mercedes de la Cruz, Bastien Cautain, Maria Cândida Monteiro, Fernando Reyes, Laurent Rios, Thierry Perez, Olivier P. Thomas

**Affiliations:** 1 Nice Institute of Chemistry UMR 7272 CNRS—PCRE, University of Nice-Sophia Antipolis, Parc Valrose, 06108 Nice, France; E-Mails: coralie.audoin@unice.fr (C.A.); dominique.bonhomme@unice.fr (D.B.); julijana@scripps.edu (J.I.); 2 GREENSEA SAS, Promenade du Sergent Jean-Louis Navarro, 34140 Mèze, France; E-Mail: laurentrios@greentech.fr; 3 Institut Méditerranéen de Biodiversité et d’Ecologie, Aix-Marseille University, UMR 7263 CNRS, Station Marine d’Endoume, 13007 Marseille, France; E-Mail: thierry.perez@imbe.fr; 4 Fundación MEDINA, Centro de Excelencia en Investigación de Medicamentos Innovadores en Andalucía, Avda. Del Conocimiento, 3, Parque Tecnológico de Ciencias de la Salud, 18100 Armilla, Granada, Spain; E-Mails: mercedes.delacruz@medinaandalucia.es (M.C.); bastien.cautain@medinaandalucia.es (B.C.); mcandida.monteiro@medinaandalucia.es (M.C.M.); fernando.reyes@medinaandalucia.es (F.R.)

**Keywords:** sponge, *Oscarella*, Homoscleromorpha, sesterterpenes, saponins

## Abstract

The chemical investigation of the recently described Mediterranean Homoscleromorpha sponge *Oscarella balibaloi* revealed an original family of five closely related glucosylated sesterterpenes **1**–**4**, named balibalosides. Their structure elucidation was mainly inferred from NMR and HRMS data analyses. Balibalosides differ by the pattern of acetyl substitutions on the three sugar residues linked to the same aglycone sesterterpenoid core. From a biosynthetic perspective, these compounds may represent intermediates in the pathways leading to more complex sesterterpenes frequently found in Dictyoceratida, a sponge Order belonging to Demospongiae, a clade which is phylogenetically distinct from the Homoscleromorpha. While steroid and triterpenoid saponins were already well known from marine sponges, balibalosides are the first examples of glycosilated sesterterpenes.

## 1. Introduction

The Mediterranean Sea is characterized by a high number of habitats which created and maintain a great diversity of living forms, making this biogeographic part of the oceans a renowned hotspot for biodiversity. Among rocky substrate communities, marine caves are an example of poorly investigated shallow habitat. The most recent inventory of biodiversity presents these typical geomorphological habitats as a reservoir of an exceptional richness in sessile invertebrates [1]. In caves, sponges are often the predominant organisms, both in terms of biomass and diversity. Among sponges commonly encountered in marine caves, our attention has been given to Homoscleromorpha, one of the four classes of Porifera [2], which represents only 3% of the Mediterranean sponge diversity [3], but also one of the highest rates of description of new species during the past 20 years [4]. Homoscleromorpha taxonomy is challenging due to the lack of clear morphological characters [2,4,5], although the knowledge of this group has been improved by genetic studies combined with the identification of specific cytological and chemical markers (for reviews see [3,4]). Recently, an untargeted metabolomics approach has been developed to help in the species identification and classification of Mediterranean Homoscleromorpha species [6]. This method has been proposed within an integrative taxonomy approach, today considered the most reliable and efficient way to evaluate the status of a species [4]. We therefore demonstrated that sponge metabolites, though mostly explored for their medicinal potential, are also of great interest as complementary tools for the resolution of species-complexes, and even to support phylogenetic hypotheses [7,8,9]. Finally, the untargeted metabolomics approach, designed to simultaneously measure as many metabolites as possible without bias, has a great potential to reveal unknown metabolites, which have not yet been described and/or have never been assigned to a given species or group of organisms [10].

**Figure 1:**
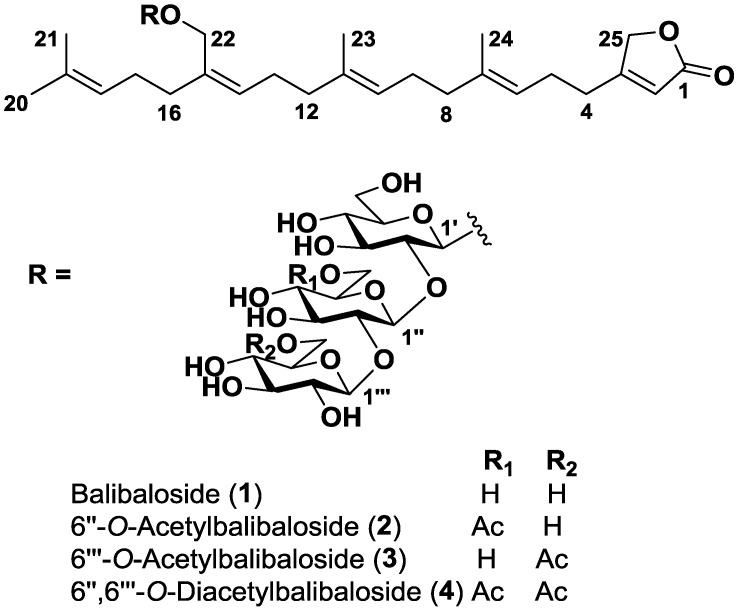
Chemical structures of balibalosides **1**–**4**.

The chemical diversity of Homoscleromorpha sponges has been poorly studied, with the notable exceptions of several species of the genus *Plakortis* (Family Plakinidae). This genus appeared as a prolific source of oxidized polyketides among which endoperoxides are now accepted as compounds with a real pharmacological potential [11,12]. In contrast, within the genus *Oscarella* (Family Oscarellidae) only two Mediterranean sister species *Oscarella lobularis* and *O. tuberculata* have been studied. Lysophospholipids, lyso-PAF and LPE C20:2, were identified as their major shared metabolites and 5-alkylpyrrole aldehydes as metabolic markers specific to *Oscarella tuberculata* [8,13,14,15]. *Oscarella balibaloi* is a recently described species from the NW Mediterranean, which tends to become more abundant in numerous marine caves across the Mediterranean Sea [9]. In a preliminary approach, the metabolic fingerprint of *O. balibaloi* showed unique high metabolite diversity when compared to the fingerprints of other *Oscarella* species. We therefore decided to undertake the isolation and structure identification of the main secondary metabolites produced by this species. We report herein the isolation and structure identification of a new family of simple glucosylated sesterterpenes **1**–**4**, named balibalosides, which differ mainly by the pattern of acetyl substitutions on the sugar residues (Figure 1).

## 2. Results and Discussion

After a CH_2_Cl_2_/MeOH (1:1) extraction of a freeze-dried and ground sample of *O. balibaloi*, the resulting crude oil was first fractionated by reversed phase Vacuum Liquid Chromatography with solvents of decreasing polarity (H_2_O–MeOH–CH_2_Cl_2_). The MeOH fraction, which showed a promising HPLC-DAD-ELSD chromatogram profile, was further subjected to successive reversed phase HPLC purifications to yield the pure compounds **1**–**4**.

### 2.1. Structure Identification

**Figure 2:**
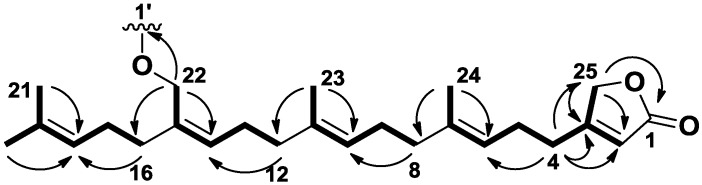
Key COSY and HMBC correlations for the aglycone of **1**–**4**.

The molecular formula of **1** was determined as C_43_H_68_O_18_ by HRESIMS ([M + Na]^+^ 895.42493). First, the ^1^H and HSQC NMR spectra of **1** evidenced the presence of three connected hexoses associated to three anomeric protons with signals at δ_H_ 4.36 (d, *J* = 7.3 Hz, 1H, H-1′) and δ_C_ 101.3 (CH, C-1′), δ_H_ 4.71 (d, *J* = 7.9 Hz, 1H, H-1″) and δ_C_ 103.8 (CH, C-1″), and δ_H_ 4.60 (d, *J* = 7.7 Hz, 1H, H-1″′) and δ_C_ 106.2 (CH, C-1″′) (Table 1). Because these three sugar residues accounted for 18 carbons, the resulting 25 carbons could correspond to a sesterterpene aglycone. The presence of four signals at δ_H_ 1.69 (s, 3H, H-20), 1.63 (s, 3H, H-21), 1.62 (s, 3H, H-23) and 1.65 (s, 3H, H-24), corresponding to methyls linked to a carbon-carbon double bond, was consistent with a terpenoid origin of the aglycone (Table 2). In addition to the four trisubstituted double bonds commonly found in terpenoids with signals at δ_C_ 123.9 (CH, C-6), 138.1 (C, C-7), 125.7 (CH, C-10), 135.8 (C, C-11), 130.8 (CH, C-14), 136.5 (C, C-15), 125.7 (CH, C-18), and 132.2 (C, C-19), the ^13^C and HSQC NMR spectra of **1** highlighted the presence of a β-substituted and α,β-unsaturated γ-butyrolactone with characteristic signals at δ_C_ 177.0 (C, C-1), 115.7 (CH, C-2), 174.2 (C, C-3), and 75.0 (CH_2_, C-25) [16]. This ending part of the molecule was ascertained by the H_2_-25/C-1/C-2/C-3 HMBC correlations (Figure 2). Additional H_2_-4/C-3/C-2/C-25 HMBC correlations located the branched isoprenyl chain at C-3 of the butyrolactone. While a linear and regular tetra-isoprenyl chain would have provided five methyl singlets in the ^1^H NMR spectrum, we observed only four carbon carbon double bond substituted methyls and therefore concluded that one of the methyl was functionalized. The presence of an oxymethylene was evidenced by the characteristic signals at δ_H_ 4.32 (s, 2H, H-22) and δ_C_ 67.0 (CH_2_, C-22) which showed clear H_2_-22/C-14/C-15/C-16 HMBC correlations with the isoprenyl chain. Because of an unfortunate overlapping between signals of H_2_-9 and H_2_-17 at δ_H_ 2.12 but also H-10 and H-18 at δ_H_ 5.12 in the ^1^H NMR spectrum of **1**, the location of the substituted terpenoid unit proved to be troublesome. This uncertainty was removed using long range HMBC correlations relayed by the resolved methylene and methyl signals at δ_H_ 4.32 (s, 2H, H_2_-22) and 1.62 (s, 3H, H_3_-23) respectively (Figure 2). The stereochemistry of the three double bonds at C-6, C-10 and C-14 was assigned as *E*, *E*, and *Z*, respectively, by comparison of the ^13^C NMR chemical shifts corresponding to the allylic carbons with literature data [16].

An additional H_2_-22/C-1′ HMBC correlation connected the sesterterpene aglycone to the sugar residues. The three residues were identified as glucose derivatives on the basis of both the presence of three methyleneoxy signals reminiscent of hexopyranosyl units and the large coupling constant values (from 7 to 9 Hz) of well resolved signals for the osidic part of the molecule which were only consistent with axial/axial relative couplings of glucopyranosyl units (Table 1). The connections between the three sugar residues were easily located at C-2′ and C-2″ due to the deshielding of the corresponding carbons at δ_C_ 83.8 (CH, C-2′) and 85.2 (CH, C-2″) and also the key H-1″/C-2′ and H-1″′/C-2″ HMBC correlations. A d absolute configuration for all sugar residues was determined using combined gas chromatography/mass spectrometry (GC/MS) on *O*-trimethylsilylated and 2-butylated derivatives of the monosaccharides produced from the sample after acidic hydrolysis [17].

The molecular formula of **2** and **3** were determined as C_45_H_70_O_19_ by HRESIMS, which suggested the presence of an additional acetyl in comparison to **1** for both isomers. This assumption was further confirmed by inspection of their ^1^H NMR spectra, which exhibited the corresponding methyl signals at δ_H_ 2.04 (s, 3H, 6″-*O*-Ac) for **2**, and δ_H_ 2.10 (s, 3H, 6″′-*O*-Ac) for **3**. For compound **2**, the acetyl was located on the primary alcohol of the second glucose residue because of the deshielding of the H_2_-6″ signals at δ_H_ 4.31 (br d, *J* = 12.0 Hz, 1H, H-6″a) and 4.20 (dd, *J* = 12.0 and 5.0 Hz, 1H, H-6″b), while, for compound **3**, it was located on the primary alcohol of the third glucose residue because of the deshielding of H_2_-6″′ signals at δ_H_ 4.47 (dd, *J* = 12.0 and 2.0 Hz, 1H, H-6″′a) and 4.22 (dd, *J* = 12.0 and 5.0 Hz, 1H, H-6″′b).

The molecular formula of **4** was determined as C_47_H_72_O_20_ by HRESIMS, which suggested the presence of two additional acetyls in comparison to **1**. Both acetyls were easily located at *O*-6″ and *O*-6″′ based on the superimposition of the corresponding ^1^H and ^13^C NMR signals observed for **2** and **3**.

**Table 1:** NMR data (500 MHz, CD_3_OD) for the sugar residues of balibalosides **1**–**4**.

Residue	Position	1		2		3		4	
		δ_C_	δ_H_, mult. (*J* in Hz)	δ_C_	δ_H_, mult. (*J* in Hz)	δ_C_	δ_H_, mult. (*J* in Hz)	δ_C_	δ_H_, mult. (*J* in Hz)
Glu-1	1′	101.3	4.36, d (7.3)	101.3	4.35, d (7.6)	101.3	4.36, d (8.0)	101.3	4.34, d (8.0)
	2′	83.8	3.37, dd (9.1, 7.3)	84.9	3.34, m	83.9	3.36, m	85.0	3.30, m
	3′	77.9	3.64, t (9.1)	77.7	3.64, t (9.1)	77.8	3.56, t (9.0)	77.5	3.57, t (9.0)
	4′	70.8	3.35, m	70.9	3.36, m	70.9	3.36, m	70.9	3.35, m
	5′	77.9	3.24, m	77.9	3.24, ddd (9.3, 5.4, 2.3)	78.1	3.24, m	77.8	3.24, m
	6′	62.7	3.86, dd (11.8, 2.1)	62.7	3.86, dd (11.9, 2.3)	62.7	3.86, dd (11.9, 2.3)	62.5	3.85, dd (11.9, 2.3)
			3.63, dd (11.8, 5.6)		3.69, dd (11.9, 5.6)		3.69, dd (11.9, 5.6)		3.68, dd (11.9, 5.6)
Glu-2	1″	103.8	4.71, d (7.9)	104.2	4.65, d (7.8)	103.6	4.69, d (7.7)	103.8	4.62, d (8.0)
	2″	85.2	3.40, dd (9.1, 7.9)	84.8	3.44, dd (9.1, 7.8)	84.8	3.39, dd (9.0, 7.7)	84.6	3.41, dd (9.1, 7.8)
	3″	77.8	3.56, t (9.1)	77.7	3.55, t (9.1)	77.8	3.56, t (9.0)	77.5	3.55, t (9.1)
	4″	71.4	3.29, dd (9.1, 7.4)	71.4	3.30, m	71.6	3.30, m	71.2	3.30, m
	5″	77.9	3.24, m	75.1	3.41, m	78.0	3.24, m	75.0	3.41, m
	6″	63.0	3.80, dd (12.0, 2.3)	64.7	4.31, br d (12.0)	63.0	3.80, dd (12.0, 2.3)	64.7	4.31, br d (12.0)
			3.63, dd (12.0, 6.0)		4.20, dd (12.0, 5.0)		3.63, dd (12.0, 6.0)		4.19, dd (12.0, 5.0)
	6″-*O*-Ac			20.7	2.04, s			20.7	2.04, s
				170.5				170.5	
Glu-3	1″′	106.2	4.60, d (7.7)	106.0	4.63, d (8.0)	105.8	4.63,d (8.0)	105.8	4.66, d (8.0)
	2″′	76.3	3.27, dd (9.1, 7.7)	76.2	3.27, m	76.0	3.28, m	76.0	3.28, m
	3″′	77.6	3.38, dd (9.0, 7.8)	77.5	3.38, m	77.7	3.38, t (9.0)	77.6	3.38, t (9.0)
	4″′	71.0	3.33, m	70.9	3.35, m	71.0	3.35, m	70.9	3.35, m
	5″′	78.9	3.34, m	78.9	3.35, m	76.1	3.52, ddd (9.0, 5.0, 2.0)	76.0	3.52, ddd (9.0, 5.0, 2.0)
	6″′	62.4	3.91, dd (12.0, 1.6)	62.4	3.91, dd (12.0, 1.8)	64.3	4.47, dd (12.0, 2.0)	64.3	4.47, dd (12.0, 2.0)
			3.73, dd (12.0, 6.0)		3.73, dd (12.0, 5.0)		4.22, dd (12.0, 5.0)		4.22, dd (12.0, 5.0)
	2″′-*O*-Ac								
							
	6″′-*O*-Ac					21.0	2.10, s	21.0	2.10, s
						170.5		170.5	

**Table 2:** NMR data (500 MHz, CD_3_OD) for the aglycone of balibalosides **1**–**4**.

Position	δ_C_, mult.	δ_H_, mult. (*J* in Hz)
1	177.0, C	
2	115.7, CH	5.87, quint (1.6)
3	174.2, C	
4	29.5, CH_2_	2.52, br t (7.2)
5	26.7, CH_2_	2.34, q (7.2)
6	123.9, CH	5.17, br t (7.2)
7	138.1, C	
8	40.7, CH_2_	2.04, br t (7.4)
9	27.5, CH_2_	2.12, m
10	125.7, CH	5.12, t (7.0)
11	135.8, C	
12	40.9, CH_2_	2.02, m
13	27.2, CH_2_	2.19, br q (6.8)
14	130.8, CH	5.39, t (7.2)
15	136.5, C	
16	35.9, CH_2_	2.13, m
17	27.8, CH_2_	2.12, m
18	125.7, CH	5.12, t (7.0)
19	132.2, C	
20	26.0, CH_3_	1.69, s
21	18.6, CH_3_	1.63, s
22	67.0, CH_2_	4.32, s ^a^
23	16.2, CH_3_	1.62, s
24	16.2, CH_3_	1.65, s
25	75.0, CH_2_	4.85 ^b^

^a^ Becomes a AB system at 4.31 (d, 11.8) and 4.33 (d, 11.8) for balibalosides **2**–**4**; ^b^ Overlapped with H_2_O.

### 2.2. Biosynthetic Considerations

**Scheme 1:**
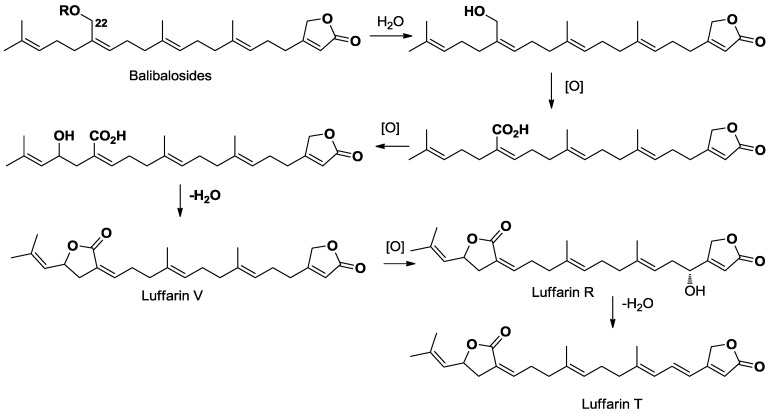
Biosynthetic considerations linking balibalosides to luffarins.

Glycosylated terpenoids are secondary metabolites increasingly found in marine sponges [18]. Among terpenoid compounds, triterpenoid and steroid saponins are the most recurring bioactive metabolites isolated from sponges. All marine organisms assembled, sesterterpenoids are considerably less distributed, with the exception of sponges of the order Dictyoceratida and marine fungi. These compounds display a great structural diversity counting linear, monocyclic, bicyclic, tricyclic and even tetracyclic derivatives [19]. Several compounds of this family have exhibited valuable bioactivity like manoalide and its derivatives, well known antibacterial agents isolated from several species of the family Thorectidae (Dictyoceratida) [20]. Despite the increasing interest in this chemical family, no glycosilated sesterterpene has ever been reported so far, and therefore balibalosides represent the first examples of such a structural class. This finding is even more intriguing as these compounds are not found in Dictyoceratida, so in the Sponge Class Demospongiae, but in the Homoscleromorpha. In this sponge class, the study of Oscarellidae family has only led to the description of lysophospholipid, alkylpyrrole aldehydes and steroid derivatives. On the other hand, a very interesting family of glycosylated polyprenyl derivatives was found in the Bahamian sponge *Plakortis simplex*, belonging to the other family of Homoscleromorpha, the Plakinidae. Plakopolyprenosides and plaxylosides were described as linear prenylated derivatives, being glycosilated at one end of the chain [11,21,22]. In our case, balibalosides only include five isoprene units and the usual butyrolactone found in Dictyoceratida sesterterpenoids. Glycosilation occurred after an already known oxidation of a methyl group of the prenylated linear chain. From a biosynthetic point of view, the presence of these sesterterpenoids is of high interest as these simple compounds are undoubtedly the precursors of more complex sesterterpenes that can be found in other sponges of Dictyoceratida order. For example, some luffarin derivatives isolated from the Australian sponge *Luffariella geometrica* are strongly similar to the linear aglycone of balibalosides, being oxidized at the same C-22 position (Scheme 1) [16,23].

### 2.3. Bioassays

Compounds **1**–**5** were tested in a wide panel of biological assays, including antibacterial activity against gram positive (methicillin resistant *Staphylococcus aureus*) and gram negative (*Acinetobacter baumannii*, *Escherichia coli*, and *Pseudomonas aeruginosa*) bacteria, antifungal activity against *Candida albicans* and *Aspergillus fumigatus*, and cell growth inhibition of several tumoral cell lines, including human breast (HCC1954, Hs578t and MCF-7), human pancreas (BxPC3, MiaPaca-2 and Panc-1), and human liver (HepG2). Unfortunately, none of the compounds displayed biological activity at the highest concentration tested (50 μg/mL for the cytotoxicity assays and 160 μg/mL for the antimicrobial tests).

## 3. Experimental Section

### 3.1. General Experimental Procedures

Optical rotations were determined in MeOH at 20 °C using a Jasco T2000 polarimeter. NMR spectra were measured on a Bruker Avance 500 MHz spectrometer with pulsed field gradient and referenced to residual solvent signals (CD_3_OD, at δ_H_ 3.31 and δ_C_ 49.0 ppm). HRESIMS data were measured with a LTQ Orbitrap mass spectrometer (Thermo Finnigan). HPLC purification was carried out on a Waters 600 system equipped with a Waters 717 Plus autosampler, a Waters 996 photodiode array detector, and a Sedex 55 evaporative light-scattering detector (Sedere, France).

### 3.2. Animal Material

Specimens of *O. balibaloi* were sampled between 15 and 35 m depth in two sites off Marseilles where the species has been described first: Maire Island (43.2096° N, 5.3353° W), and Frioul Island (43.2802° N, 5.2862° E). A voucher specimen is kept in the Station Marine d’Endoume (Marseille, France).

### 3.3. Extraction and Isolation

The dry biomass was extracted with CH_2_Cl_2_/MeOH (1:1), and the crude extract (4.7 g) was subjected to C_18_ vacuum liquid chromatography successively eluted by H_2_O, H_2_O/MeOH (1:1), MeOH, MeOH/CH_2_Cl_2_ (1:1) and CH_2_Cl_2_. The MeOH fraction was purified by semipreparative reversed-phase HPLC (Waters XSelect phenyl-hexyl, 250 × 10 mm, 5 μm, eluting from H_2_O/ACN, 60:40 to 30:70) to afford pure compounds **1** (4.7 mg), **2** (4.2 mg), **4** (2.4 mg) and **5** (2.6 mg). Pure compound **3** (1.4 mg) was obtained after an additional purification step by semipreparative reversed-phase HPLC (Waters Symmetry C18, 300 × 7.8 mm, 7 μm eluting with isocratic conditions H_2_O/ACN 50:50).

### 3.4. Balibaloside (**1**)

Colorless oil; [α]_D_^20^ −13.2 (*c* 0.45, MeOH); UV (MeOH) λ_max_ (log ε) 239 (3.46) nm; ^1^H and ^13^C NMR see Table 1 and Table 2; IR (KBr) λ_max_ 3600–3100 (br), 1740, 1635 cm^−1^; ESIMS *m/z* 895.2 [M + Na]^+^; HRESIMS *m/z* 895.42493 [M + Na]^+^ (calcd for C_43_H_68_O_18_Na, 895.42979, Δ −5.4 ppm).

### 3.5. 6″-*O*-Acetylbalibaloside (**2**)

Colorless oil; [α]_D_^20^ −23.9 (*c* 0.42, MeOH); UV (MeOH) λ_max_ (log ε) 240 (3.65) nm; ^1^H and ^13^C NMR see Table 1 and Table 2; ESIMS *m/z* 937.1 [M + Na]^+^; HRESIMS *m/z* 937.43918 [M + Na]^+ ^(calcd for C_45_H_70_O_19_Na, 937.44035, Δ −5.5 ppm).

### 3.6. 6″′-*O*-Acetylbalibaloside (**3**)

Colorless oil; [α]_D_^20^ −8.0 (*c* 0.14, MeOH); UV (MeOH) λ_max_ (log ε) 239 (3.32) nm; ^1^H and ^13^C NMR see Table 1 and Table 2; ESIMS *m/z* 937.2 [M + Na]^+^; HRESIMS *m/z* 937.43469 [M + Na]^+^ (calcd for C_45_H_70_O_19_Na, 937.44035, Δ −6.0 ppm).

### 3.7. 6″,6″′-*O*-Diacetylbalibaloside (**4**)

Colorless oil; [α]_D_^20^ −5.4 (*c* 0.24, MeOH); UV (MeOH) λ_max_ (log ε) 239 (2.85) nm; ^1^H and ^13^C NMR see Table 1 and Table 2; ESIMS *m/z* 979.2 [M + Na]^+^; HRESIMS *m/z* 979.45038 [M + Na]^+^ (calcd for C_47_H_72_O_20_Na, 979.45092, Δ −0.5 ppm).

### 3.8. Absolute Configuration of the Sugar Residues

0.5 mg of compound **1** was placed into a test tube. To this, 400 μL of 2 M aqueous trifluoroacetic acid was added. The tube was then placed at 121 °C for 1.5 h. Once cooled, the sample was dried under nitrogen. After drying the sample was butylated using *S*-(+)-2-butanol for 16 h at 80 °C. Finally, the sample was per-*O*-trimethylsilylated by treatment with Tri-Sil (Pierce) at 80 °C (0.5 h). GC/MS analysis of the butylated derivatives was performed on an Agilent 7890A GC interfaced to a 5975C MSD, using an Agilent DB-1 fused silica capillary column (30 m × 0.25 mm ID). Individual standards of glucose were run in parallel with the sample. Comparison of retention times allowed for the elucidation of the absolute configuration of the monosaccharides [17].

### 3.9. Cytotoxicity Bioassays

All cell lines were obtained from the American Type Culture Collection (ATCC, Manassas, VA, USA). Media, serum and complements were purchased from Invitrogen. HCC1954, Hs578t cells (human breast carcinoma, CRL-2316 and HTB-126 respectively) and BxPC3 cells (human pancreas adenocarcinoma CRL-1687) were maintained in RPMI medium supplemented with 10% fetal bovine serum (FBS), 2 mM l-glutamine, 100 U/mL penicillin, and 100 μg/mL streptomycin. HepG2 cells (human liver carcinoma, CCL-8065) were grown in ATCC-formulated Eagle’s M essential medium (MEM) with 10% qualified FBS, 2 mM l-glutamine, 1 mM sodium pyruvate, and 100 μM MEM non-essential amino acids. MCF-7 cells (human breast HTB-22) were maintained in the previous medium supplemented with 0.01 mg/mL bovine insulin. MiaPaca-2 cells (human pancreas carcinoma CRL-1420) and Panc-1 (human pancreas hepitelioid carcinoma CRL-1469) were cultured routinely in Dulbecco’s Modified Eagle’s Medium (DMEM) with 10% FBS, 100 U/mL penicillin, and 100 μg/mL streptomycin. All cell cultures were maintained at 37 °C under a humidified atmosphere of 5% CO_2_. MTT reduction rate is an indicator of the functional integrity of the mitochondria and, hence, of cellular viability [24,25,26]. For the assay, the number of cells seeded per culture well was 100.000 cells/mL. A total of 5 μL of each compound titration concentration was dispensed into 400 μL of fresh medium. From this mixture, 100 μL were added to three different cell plates at a final maximum concentration of compound per well of 50 μg/mL, and 1.25% DMSO to minimize any solvent toxicity background. After 72 h treatment, MTT reduction is estimated by measuring absorbance at 590 nm. 10 dilutions 1/2 were performed in triplicate for each compound and in each cell line.

### 3.10. Antimicrobial Bioassays

Four bacterial and two fungal strains were used for the evaluation of the antimicrobial activity of the balibalosides. Antibacterial susceptibility was tested against methicillin resistant *Staphylococcus aureus* (MRSA) from MEDINA’s Culture Collection, *Acinetobacter baumannii*, a clinical isolate from MEDINA’s Culture Collection, *Escherichia coli* (envA/tolC) from MEDINA’s Culture Collection and *Pseudomonas aeruginosa* PAO-1. Antifungal susceptibility was tested against *Candida albicans*, clinical isolate from MEDINA’s Culture Collection, and *Aspergillus fumigatus* ATCC 46645 (wild-type strain).

Frozen stocks of *C. albicans* were used to inoculate Sabouraud Dextrose Agar (SDA) plates for confluent growth. Plates were incubated for 24 h, at 35 °C. The grown colonies were harvested from the SDA plates and suspended in RPMI-1640 modified medium. Modified RPMI-1640 medium was prepared as follows: 20.8 g of RPMI powder (Sigma) were poured into a 2 L flask, together with 13.4 g of YNB, 1.8 L of milliQ water, 80 mL of Hepes 1 M and 72 mL of glucose 50%. The volume was adjusted to 2 L and filtered. The OD_660_ was adjusted to 0.25 using RPMI-1640 modified as diluent and blank. This inoculum was diluted 1:10 and kept on ice until used to inoculate 96-well microtiter plates. For the assay, 90 μL/well of the 1:10 diluted inoculum were mixed with 1.6 μL/well of compound solution in DMSO and 8.4 μL/well of RPMI-1640 modified medium. Amphotericin B and Penicillin G were used as internal positive and negative controls respectively. After dispensing the inoculums, the samples and the controls, the assay plates were read in a Tecan Ultraevolution spectrophotometer at 612 nm for *T*_0_ (zero time). Then, the plates were statically incubated at 37 °C for 20 h. After incubation, the plates were shaken in a DPC Micromix-5 and read again for *T_f_* (final time). Percentage of growth inhibition was calculated using the following equation:

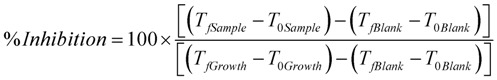


Antifungal activity against *A. fumigatus* ATCC 46645 (wild-type strain) was scored using resazurin, a non-fluorescent blue dye that after reduction is converted to the pink colored highly fluorescent resorufin. A conidial suspension was prepared from a subculture of *A. fumigatus* on PDA medium. The grown colonies were harvested from the PDA plates and suspended in RPMI-1640 modified medium. Modified medium RPMI-1640 was prepared as describes above for *C. albicans*. The inoculum concentration size was ~2.5 × 10^4^ CFU/mL (determined by counting in a Neubauer chamber) and resazurin final concentration of 0.002%. For the assay, 1 μL/well of compound solution in DMSO and 100 μL/well of the inoculum were dispensed in 96-well microtiter plates. Amphotericin B was used as positive control. After dispensing, the plates were statically incubated at 37 °C for 30 h. After incubation, the plates were read on VICTOR multilabel counter (Perkin Elmer™) using wavelength settings for resorufin (excitation 570 nm, emission 600 nm).

For the antibacterial tests, thawed stock inocula suspensions from cryovials of each microorganism (MRSA, *A. baumannii*, *E. coli* and *P. aeruginosa*) were streaked onto Luria-Bertani agar plates (LBA, 40 g/L) and incubated at 37 °C overnight to obtain isolated colonies. Single colonies of each microorganism were inoculated into 10 mL of Luria-Bertani broth medium (LB, 25 g/L in 250 mL Erlenmeyer flasks) and incubated overnight at 37 °C with shaking at 220 rpm and then diluted in order to obtain assay inocula of approximately 1.1 × 10^6^ CFU/mL (MRSA) or 5–6 × 10^5^ CFU/mL (*A. baumannii*, *E. coli*, and *P. aeruginosa*).

For the assay 90 μL/well of the diluted inoculum were mixed with 1.6 μL/well of each compound dissolved in DMSO and 8.4 μL/well of LB medium. Kanamycin and amphotericin B (MRSA), rifampicin and amphotericin B (*A. baumannii*), novobiocin and amphotericin B (*E. coli*), and ciprofloxacin and amphotericin B (*P. aeruginosa*) were included as internal plate controls. Absorbance at 612 nm was measured with a Tecan UltraEvolution spectrophotometer (Tecan, Durham, USA) at *T*_0_ (zero time) and immediately after that, plates were statically incubated at 37 °C for 20 h. After this period, the assay plates were shaken using the DPC Micromix-5 and once more the absorbance at OD 612 nm was measured at *T_f_* (final time). Percentage inhibition of growth was calculated using the same equation previously described for *C. albicans*.

Each compound was serially diluted in DMSO with a dilution factor of 2 to provide 10 concentrations starting at 160 μg/mL for all the assays. The MIC was defined as the lowest concentration of an antimicrobial or antifungal compound that inhibited ≥95% of the growth of a microorganism after overnight incubation. The data were analysed using the Genedata Screener program (Genedata AG, Switzerland). In all experiments performed in this work the RZ’ factor obtained was between 0.85 and 0.95.

## 4. Conclusions

The first chemical study of the Homoscleromorpha sponge species, *Oscarella balibaloi*, led to the isolation of five new glycosilated sesterterpenes named balibalosides. To our knowledge, they represent the first report of glycosilated sesterterpenoids from a natural source. These compounds may be biosynthetic precursors of more complex sesterterpenoids already known from another sponge group. Taking into consideration the increasing number of newly described species within Homoscleromorpha, this sponge clade represents a great potential to reveal original chemical structures. Numerous representatives of the families Oscarellidae and Plakinidae, which are confined to dark marine caves, have not been explored yet. From an integrative taxonomy and phylogeny perspective, it would be valuable to identify additional relevant metabolic synapomorphies for each identified clusters of species. This is especially true for the species *Pseudocorticium jarrei*, recently reclassified within the family Oscarellidae and as sister species of *O. balibaloi*, following the results of combined phylogenetic, cytological and untargeted metabolomics studies.

The chemical simplicity associated to the compounds produced by Oscarellidae secondary metabolites may be of high evolutionary significance. This observation can also be extended to the whole Homoscleromorpha group if we include the simple peroxides produced by species of the *Plakortis* genus. Indeed, this observation can be related with the accepted place of Homoscleromorpha in the sponge phylogenetic tree. This fourth class of marine sponges can indeed be seen as one of the most recent group of marine sponges. In the same time, the most ancient sponges of the extended class Demospongiae are usually characterized by structurally more complex compounds, apparently involving more evolved enzymatic complexes. Evidently, further investigations on the secondary metabolome of other worldwide distributed Homoscleromorpha sponges are necessary to strengthen this hypothesis.

## Supplementary Files

Supplementary File 1 Supplementary Information (PDF, 1587 KB)
