# Supplementary material for: Balibalosides, an Original Family of Glucosylated Sesterterpenes Produced by the Mediterranean Sponge Oscarella balibaloi
Source: Mar Drugs. 2013 May 6;11(5):1477–89. doi: 10.3390/md11051477 (PMC3707155; doi:10.3390/md11051477)

Supplementary Information

Table of Contents

Figure S1. ^{1}H NMR spectrum of **1** (500 MHz, CD_3OD). 2
Figure S2. ^{13}C NMR spectrum of **1** (125 MHz, CD_3OD). 3
Figure S3. COSY spectrum of **1** (500 MHz, CD_3OD). 4
Figure S4. HSQC spectrum of **1**. 5
Figure S5. HMBC spectrum of **1**. 6
Figure S6. HRESIMS of **1**. 7
Figure S7. ^{1}H NMR spectrum of **2** (500 MHz, CD_3OD). 8
Figure S8. COSY spectrum of **2** (500 MHz, CD_3OD). 9
Figure S9. HSQC spectrum of **2**. 10
Figure S10. HRESIMS of **2**. 11
Figure S11. ^{1}H NMR spectrum of **3** (500 MHz, CD_3OD). 12
Figure S12. COSY spectrum of **3** (500 MHz, CD_3OD). 13
Figure S13. HSQC spectrum of **3**. 14
Figure S14. HRESIMS of **3**. 15
Figure S15. ^{1}H NMR spectrum of **4** (500 MHz, CD_3OD). 16
Figure S16. COSY spectrum of **4** (500 MHz, CD_3OD). 17
Figure S17. HSQC spectrum of **4**. 18
Figure S18. HRESIMS of **4**. 19

Figure S1. ^{1}H NMR spectrum of **1** (500 MHz) in CD_3OD.

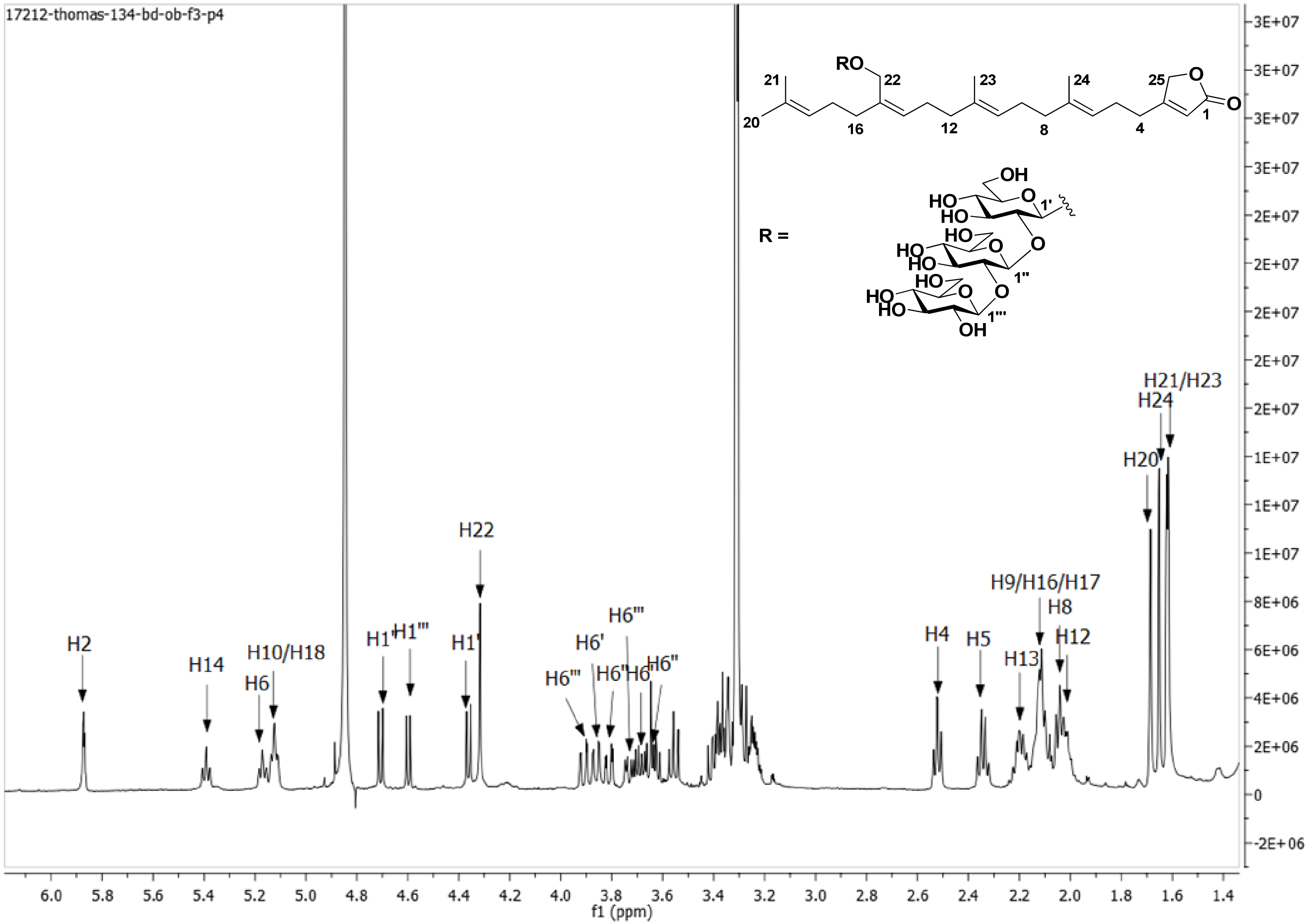

Figure S2. ^{13}C NMR spectrum of **1** (125 MHz) in CD_3OD.

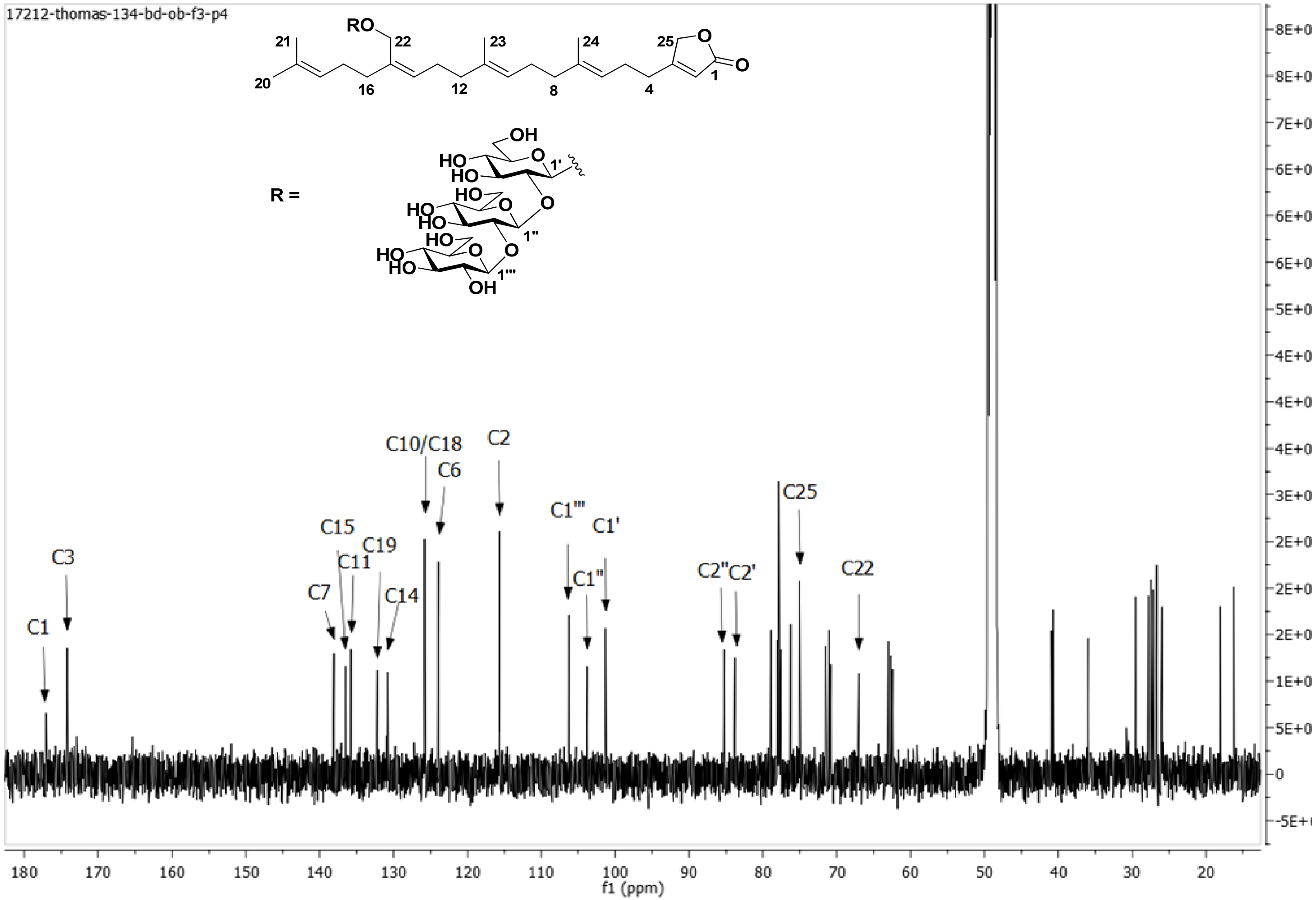

Figure S3. COSY spectrum of **1** (500 MHz) in CD_3OD.

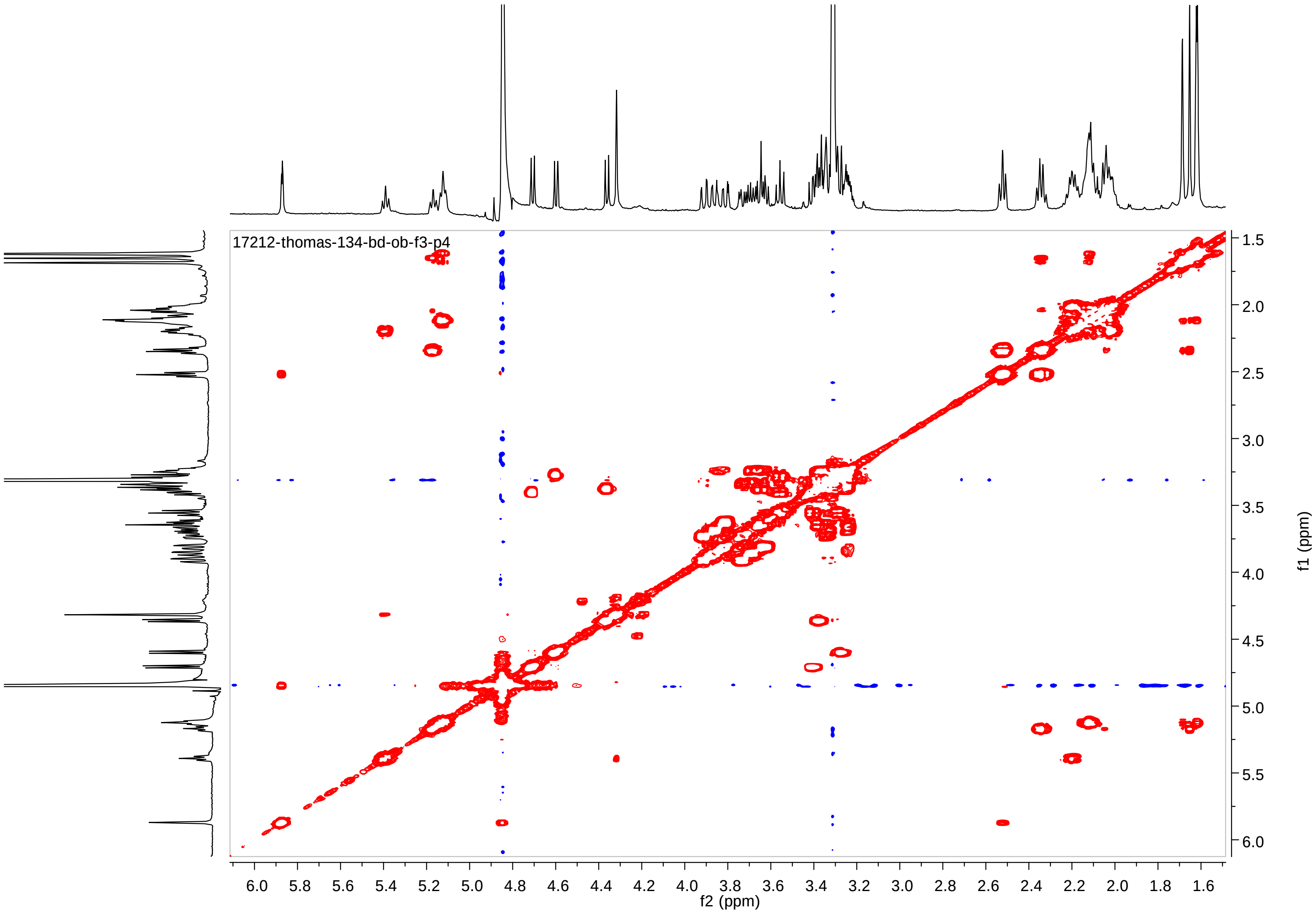

Figure S4. HSQC spectrum of **1** in CD_3OD.

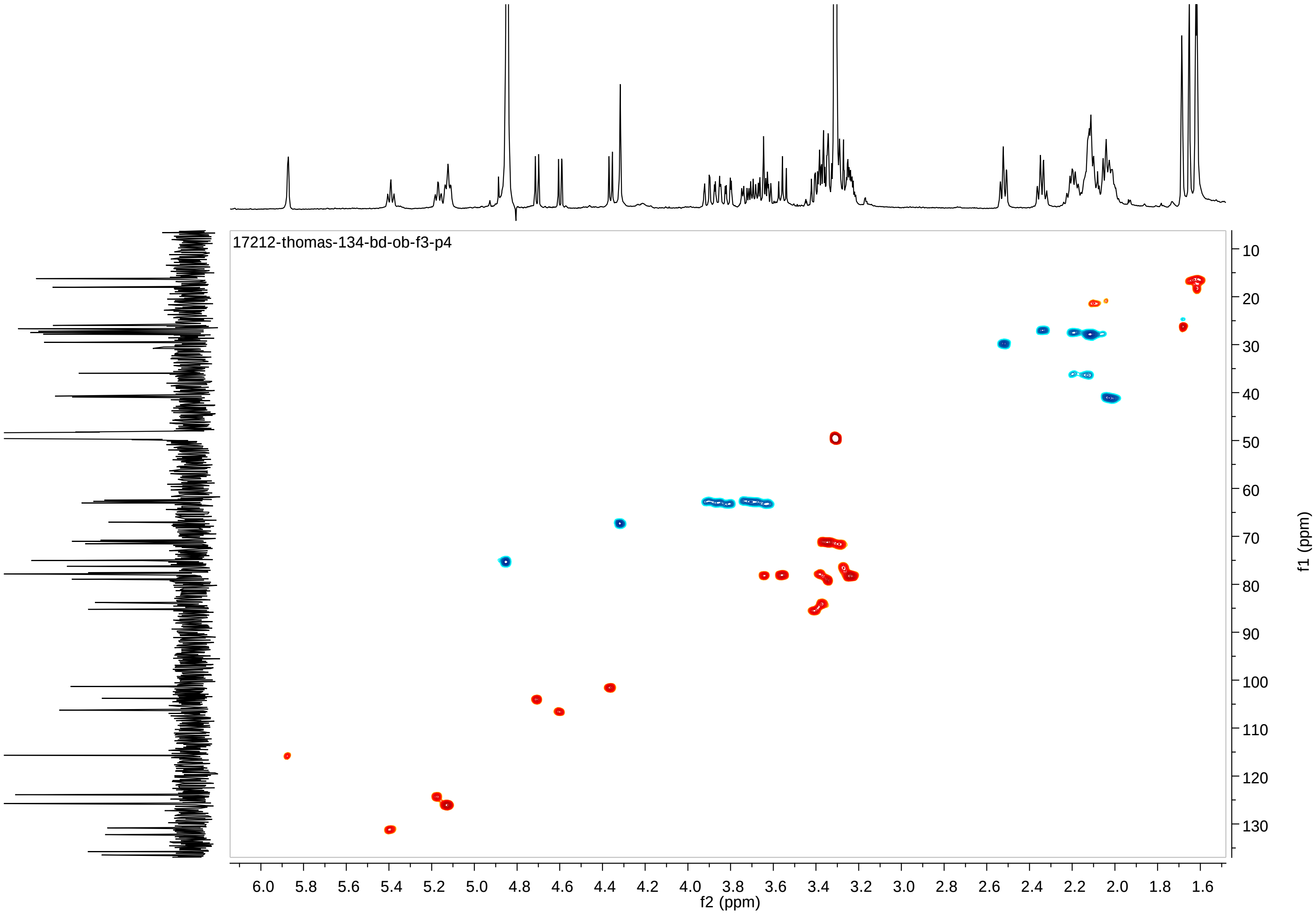

Figure S5. HMBC spectrum of **1** in CD_3OD.

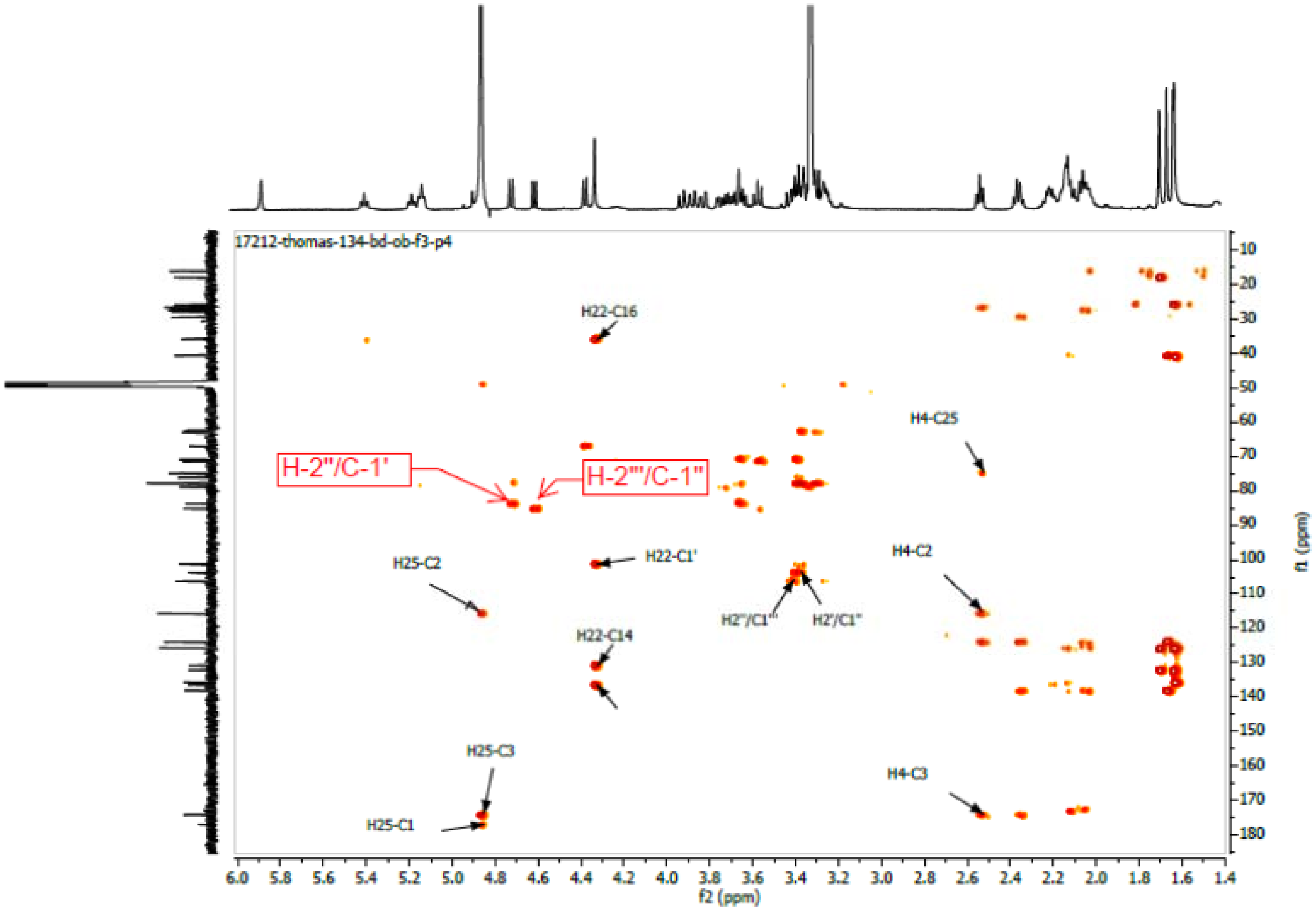

Figure S6. HRESIMS spectrum of **1**.

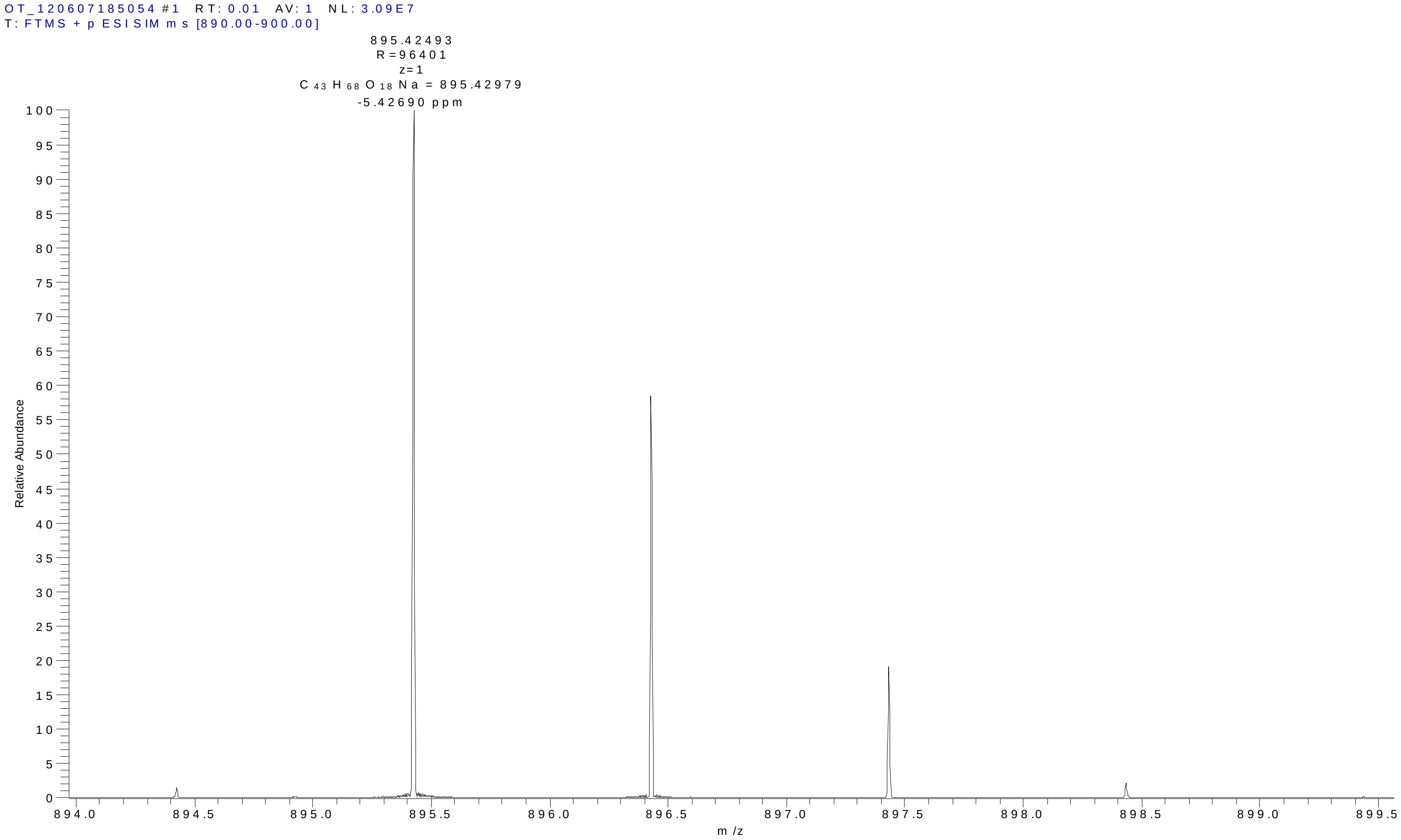

Figure S7. ^{1}H NMR spectrum of **2** (500 MHz) in CD_3OD.

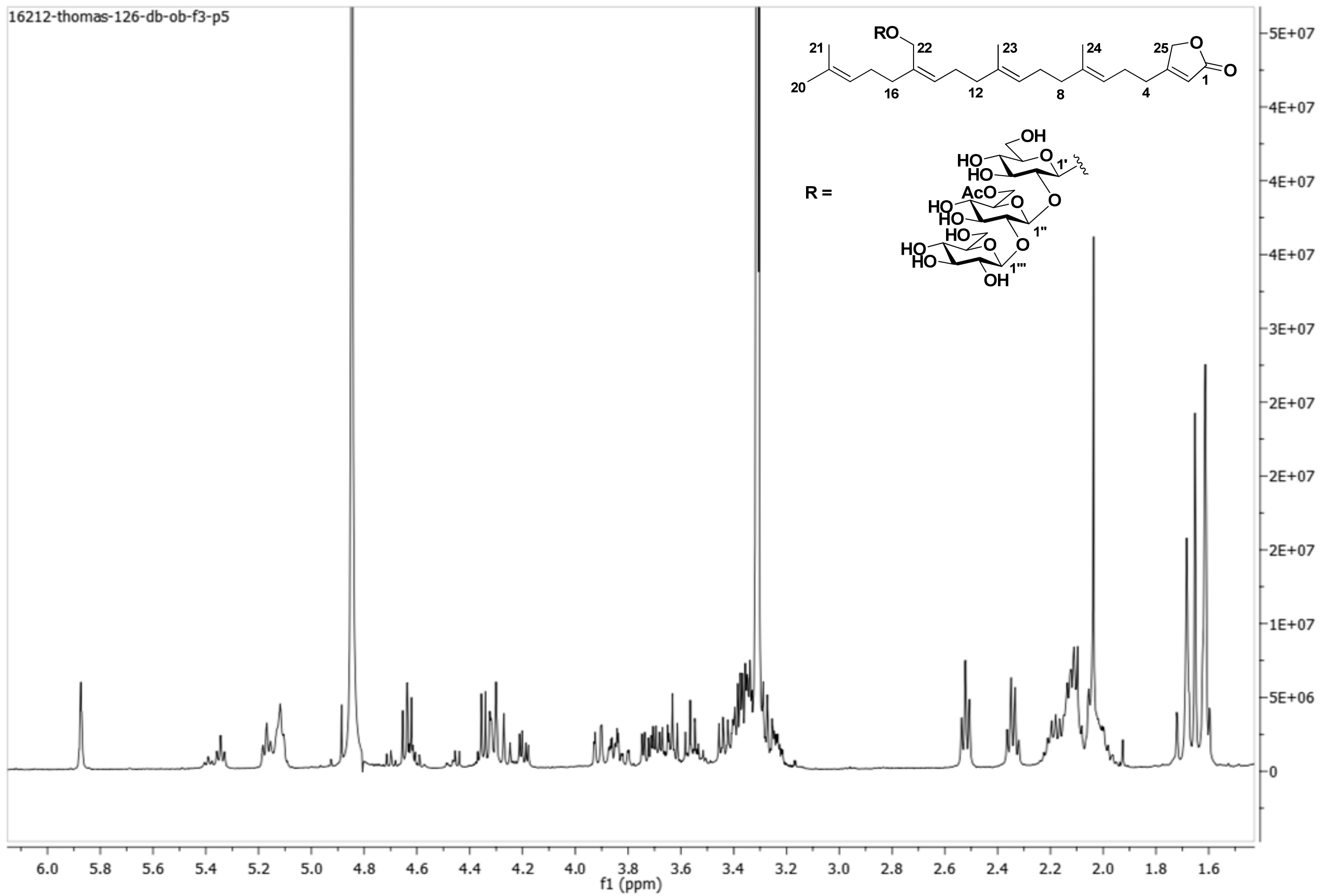

Figure S8. COSY spectrum of **2** (500 MHz) in CD_3OD.

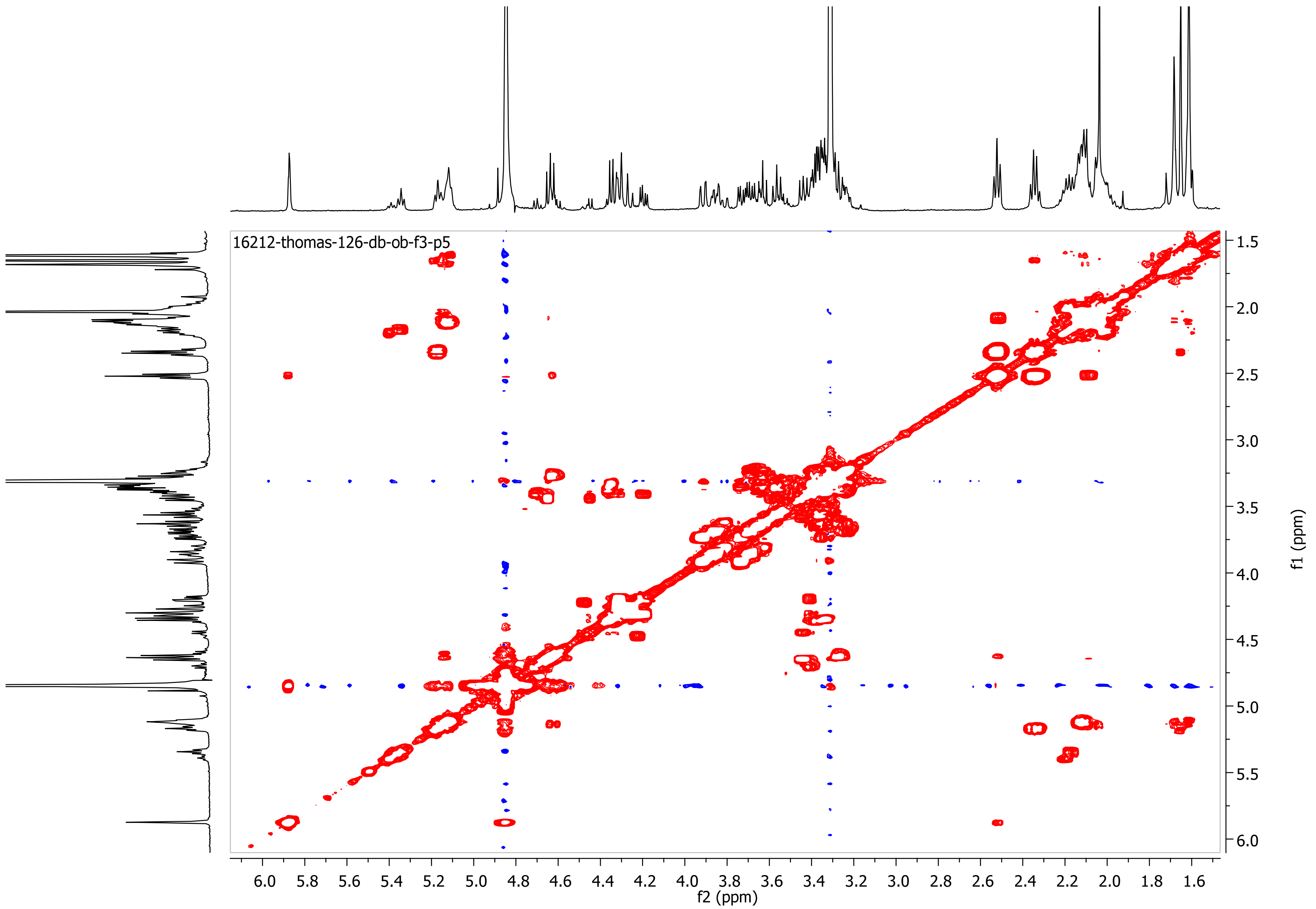

Figure S9. HSQC spectrum of **2** in CD_3OD.

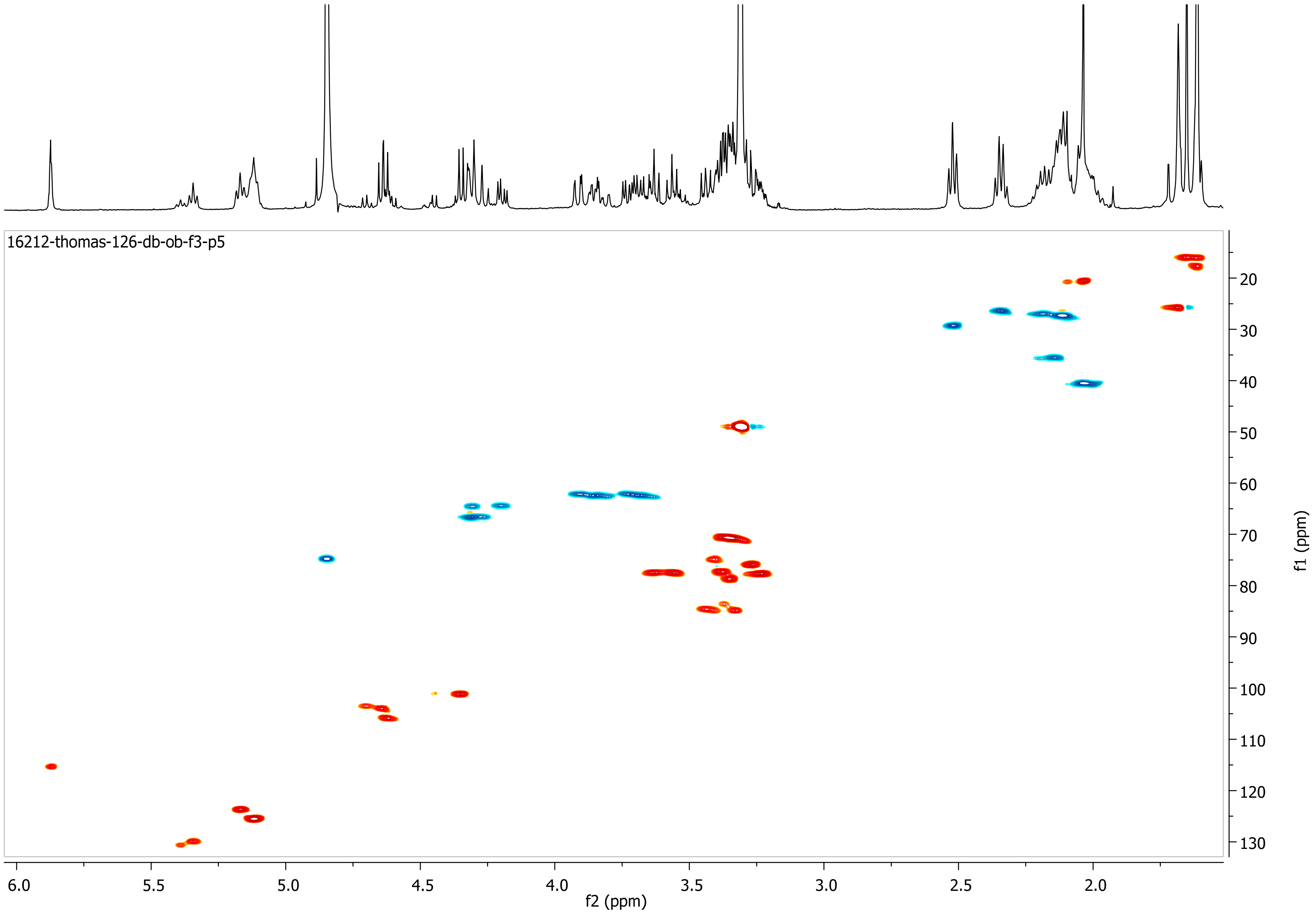

Figure S10. HRESIMS of **2**.

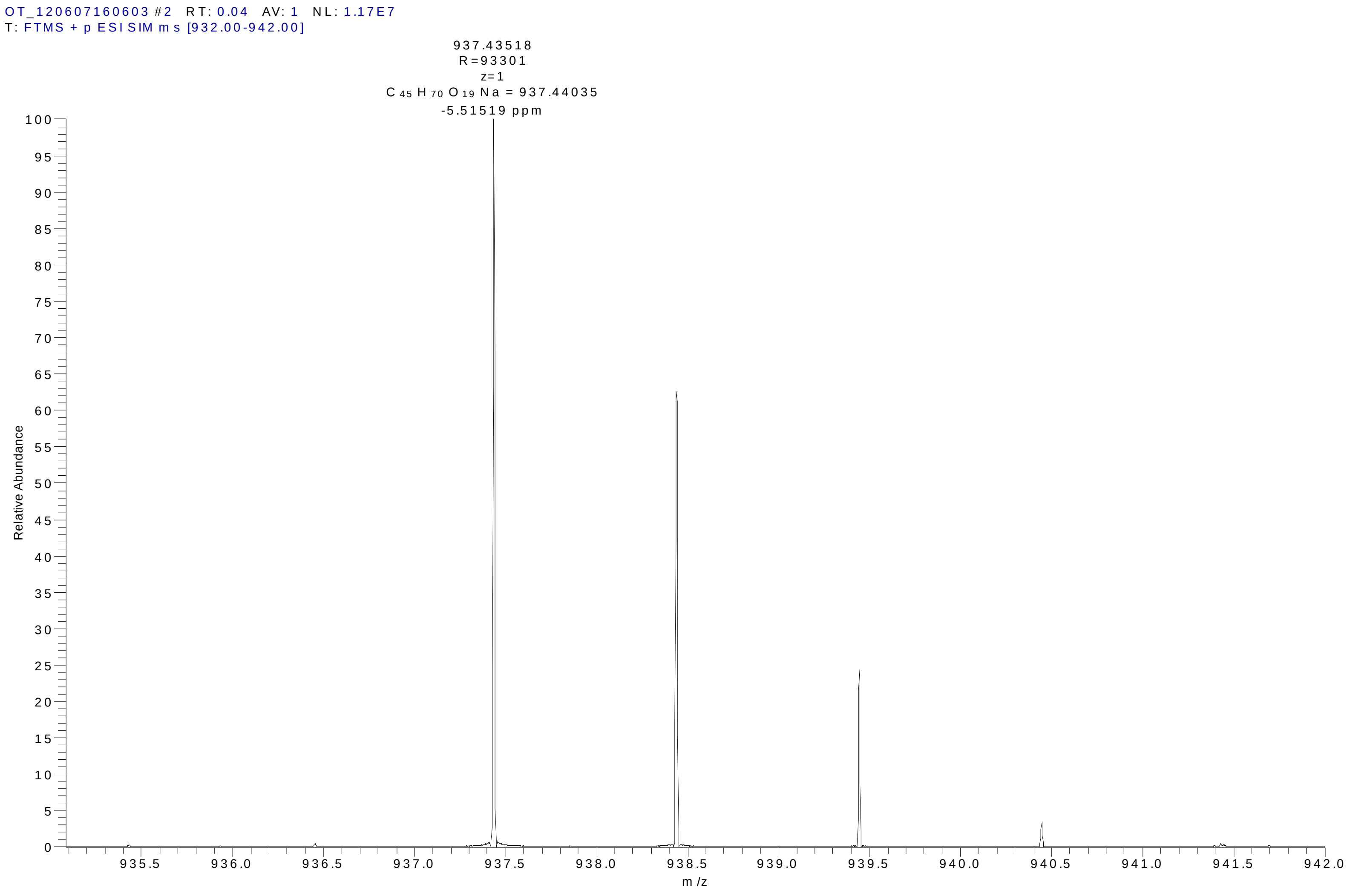

Figure S11. ^{1}H NMR spectrum of **3** (500 MHz) in CD_3OD.

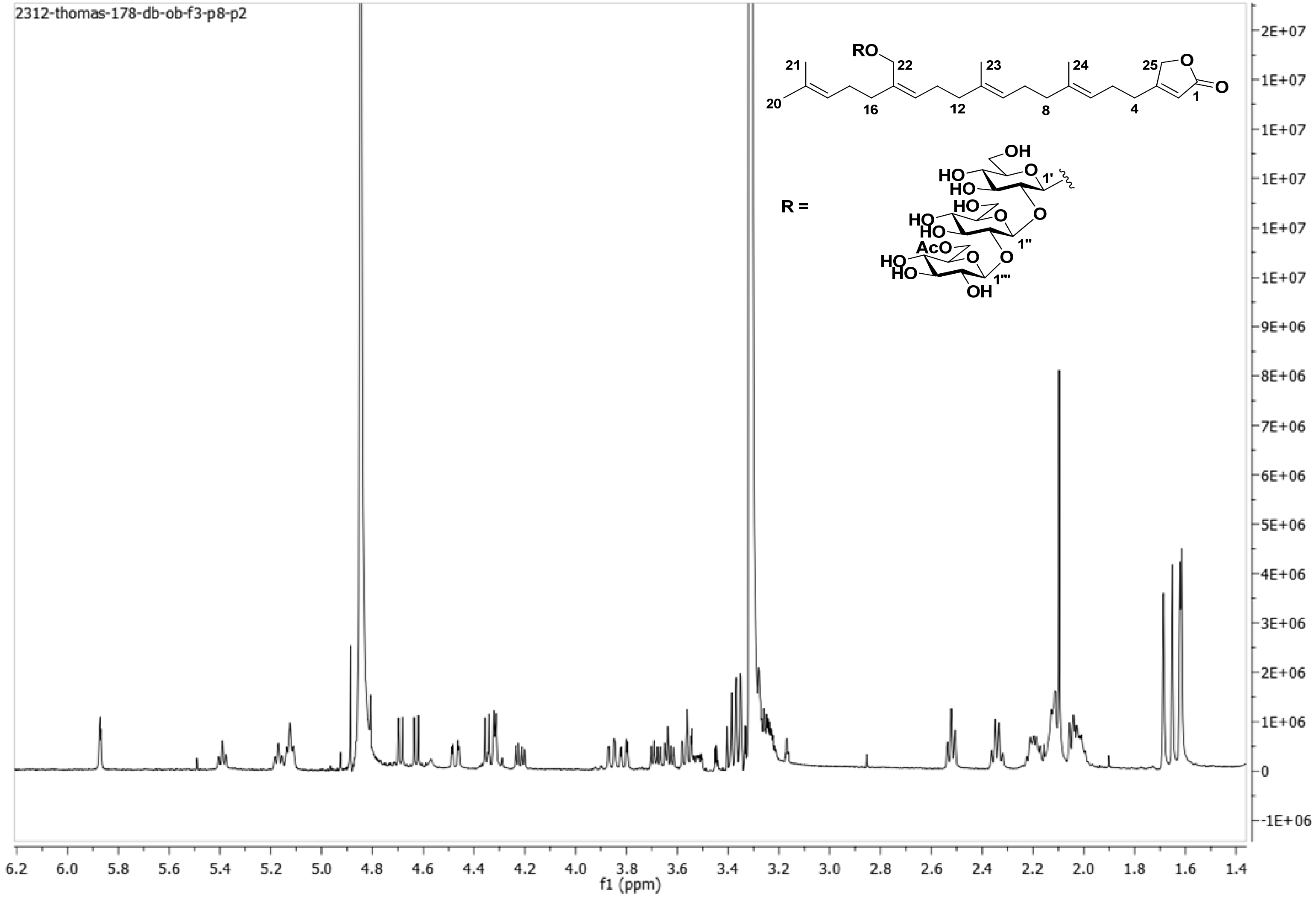

Figure S12. COSY spectrum of **3** (500 MHz) in CD_3OD.

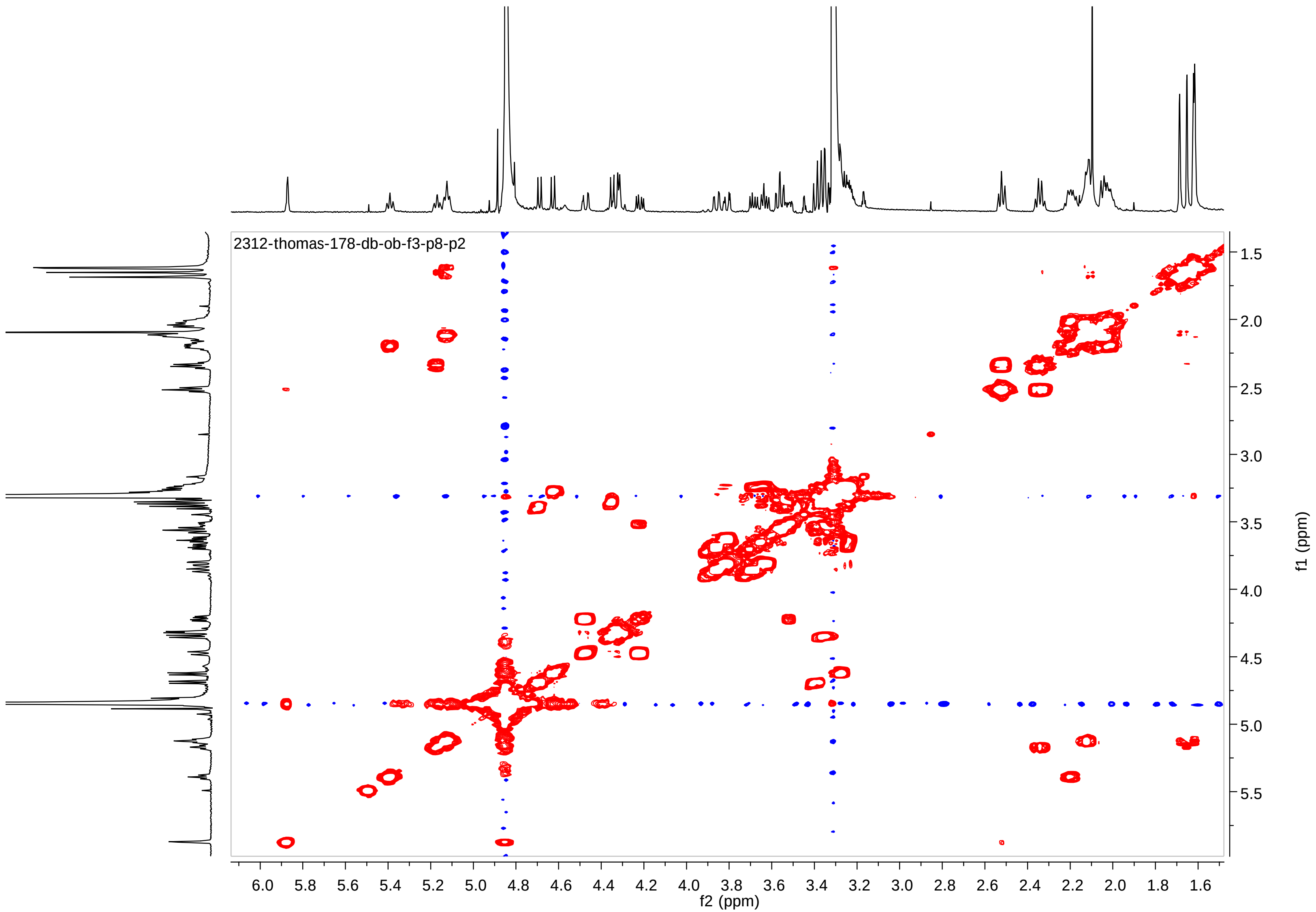

Figure S13. HSQC spectrum of **3** in CD_3OD.

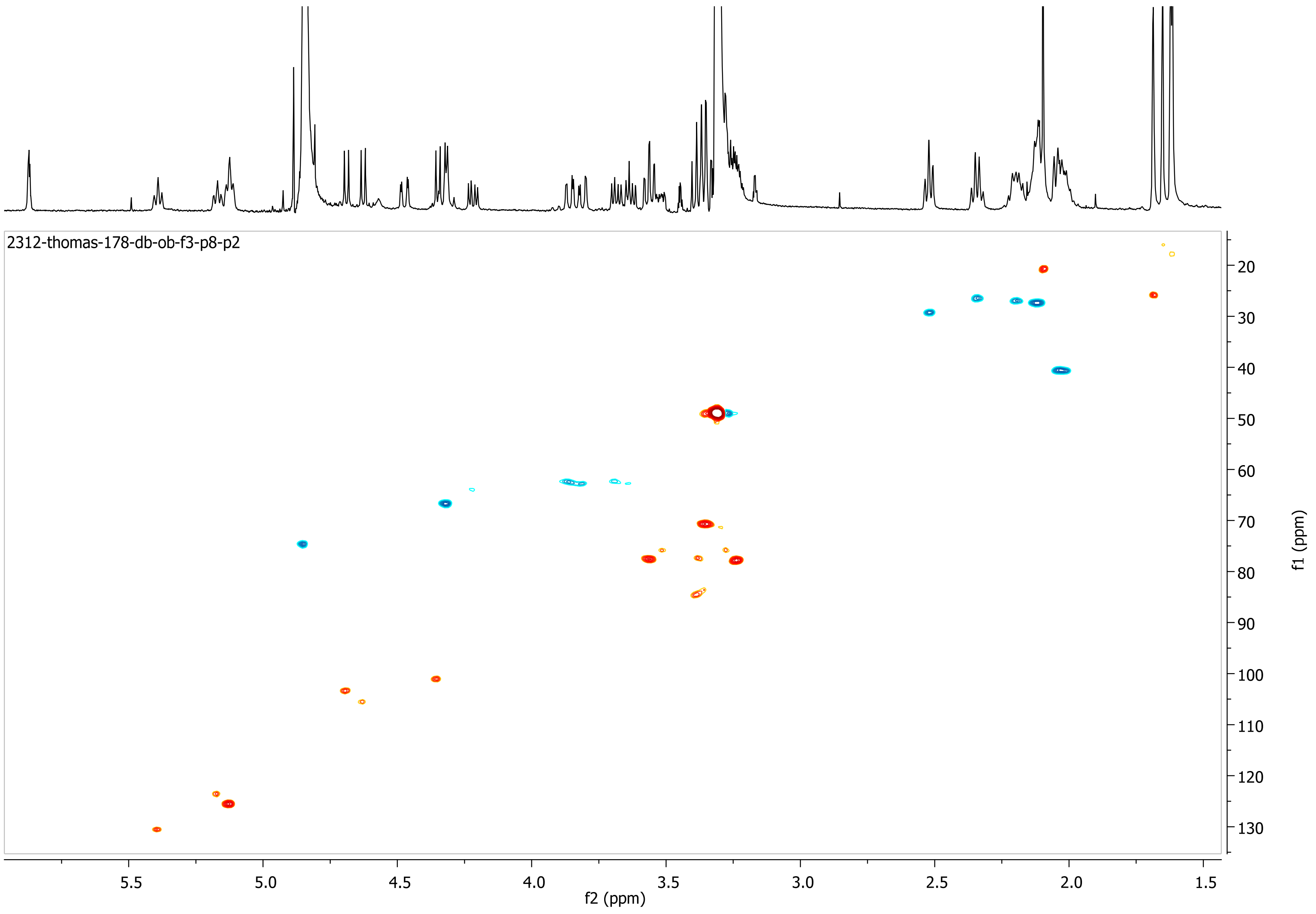

Figure S14. HRESIMS of **3**.

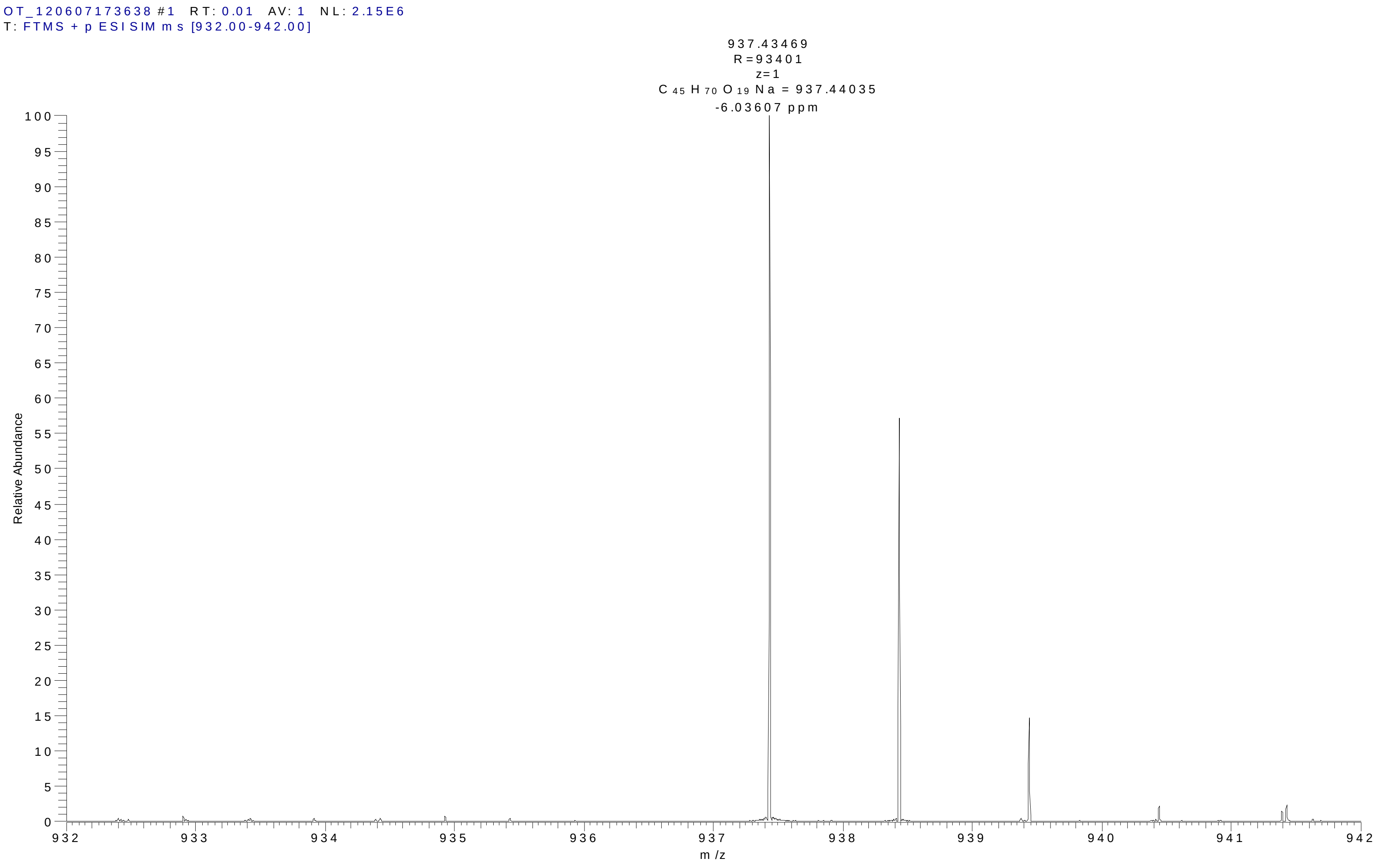

Figure S15. ^{1}H NMR spectrum of **4** (500 MHz) in CD_3OD.

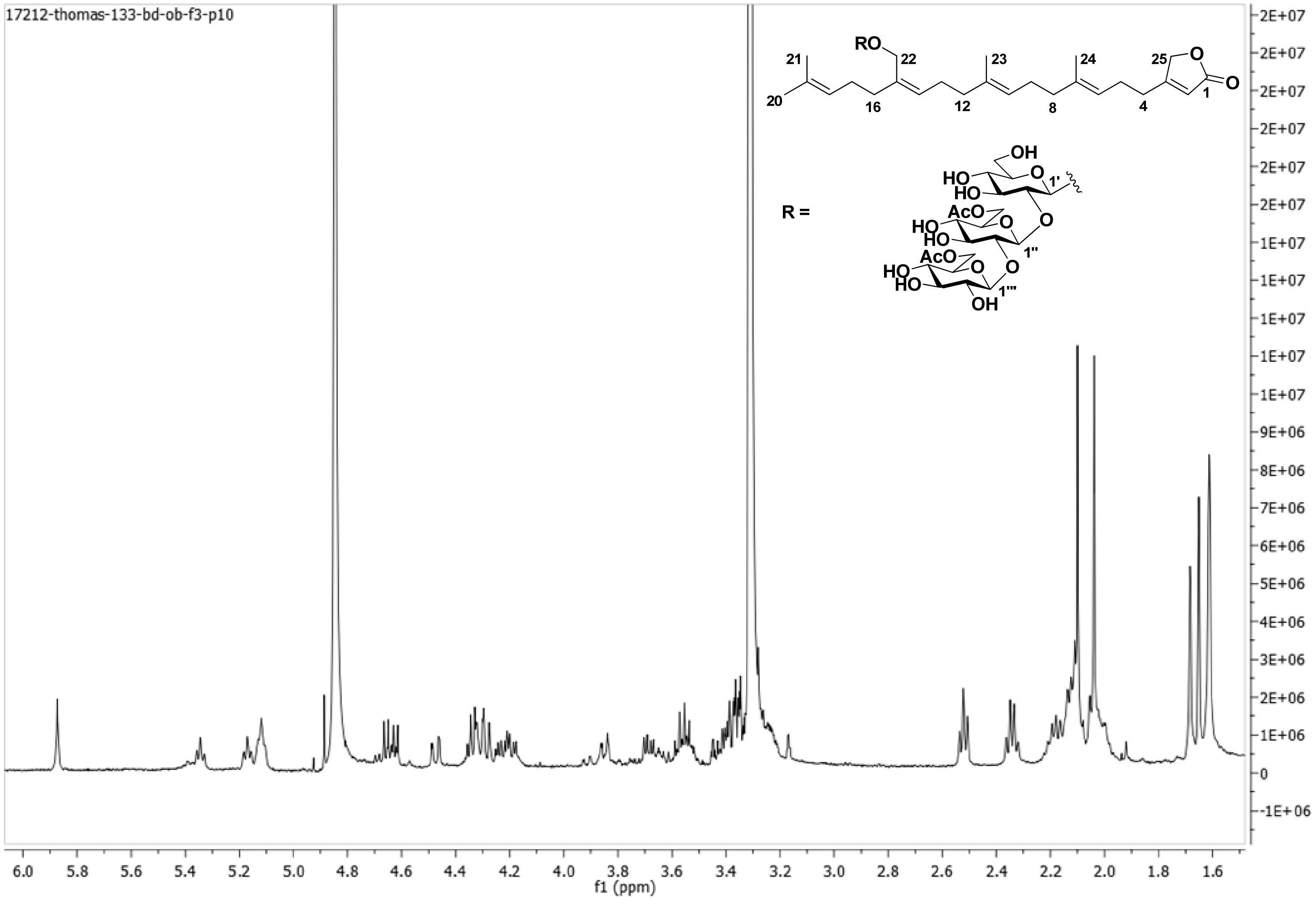

Figure S16. COSY spectrum of **4** (500 MHz) in CD_3OD.

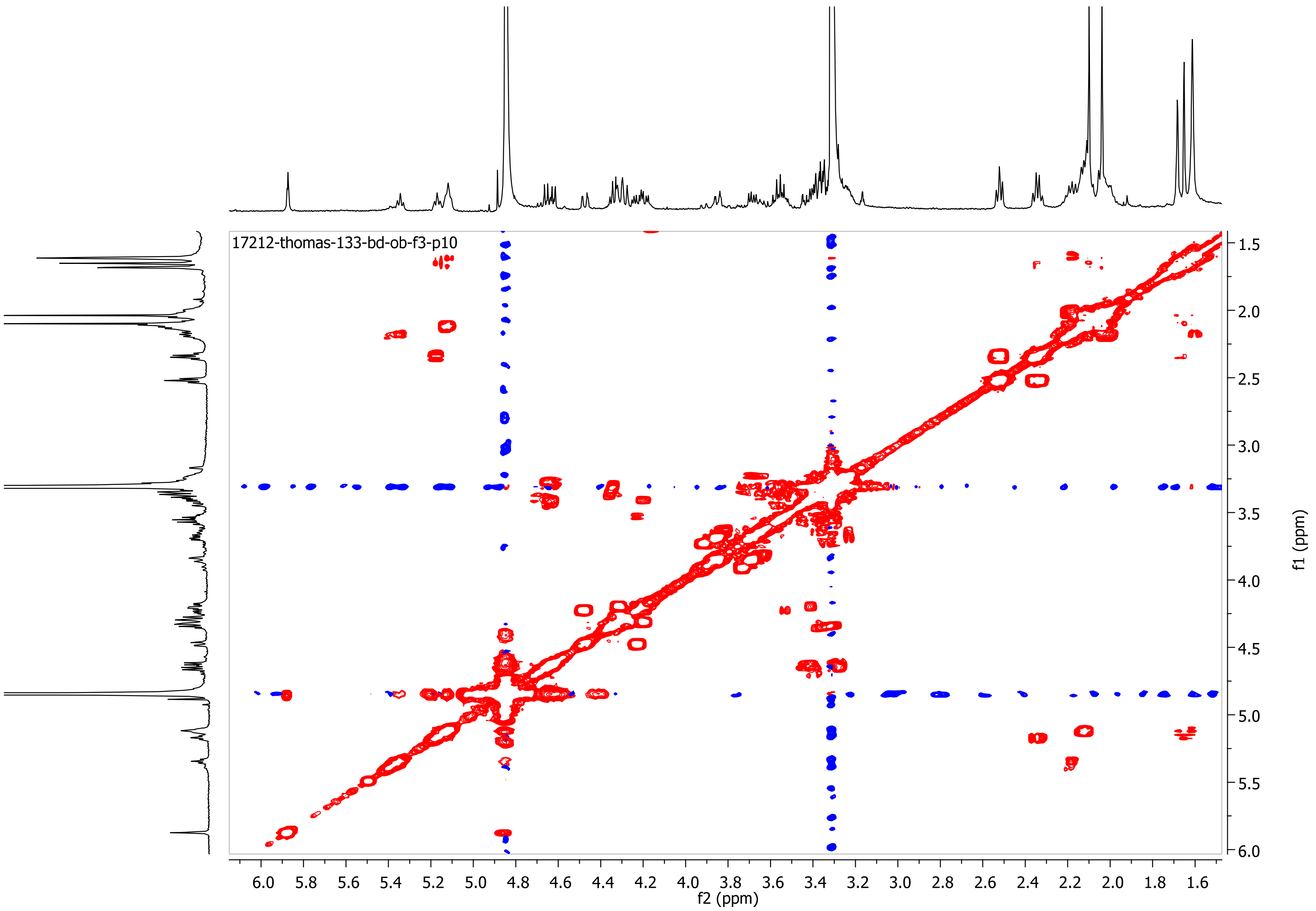

Figure S17. HSQC spectrum of **4** in CD_3OD.

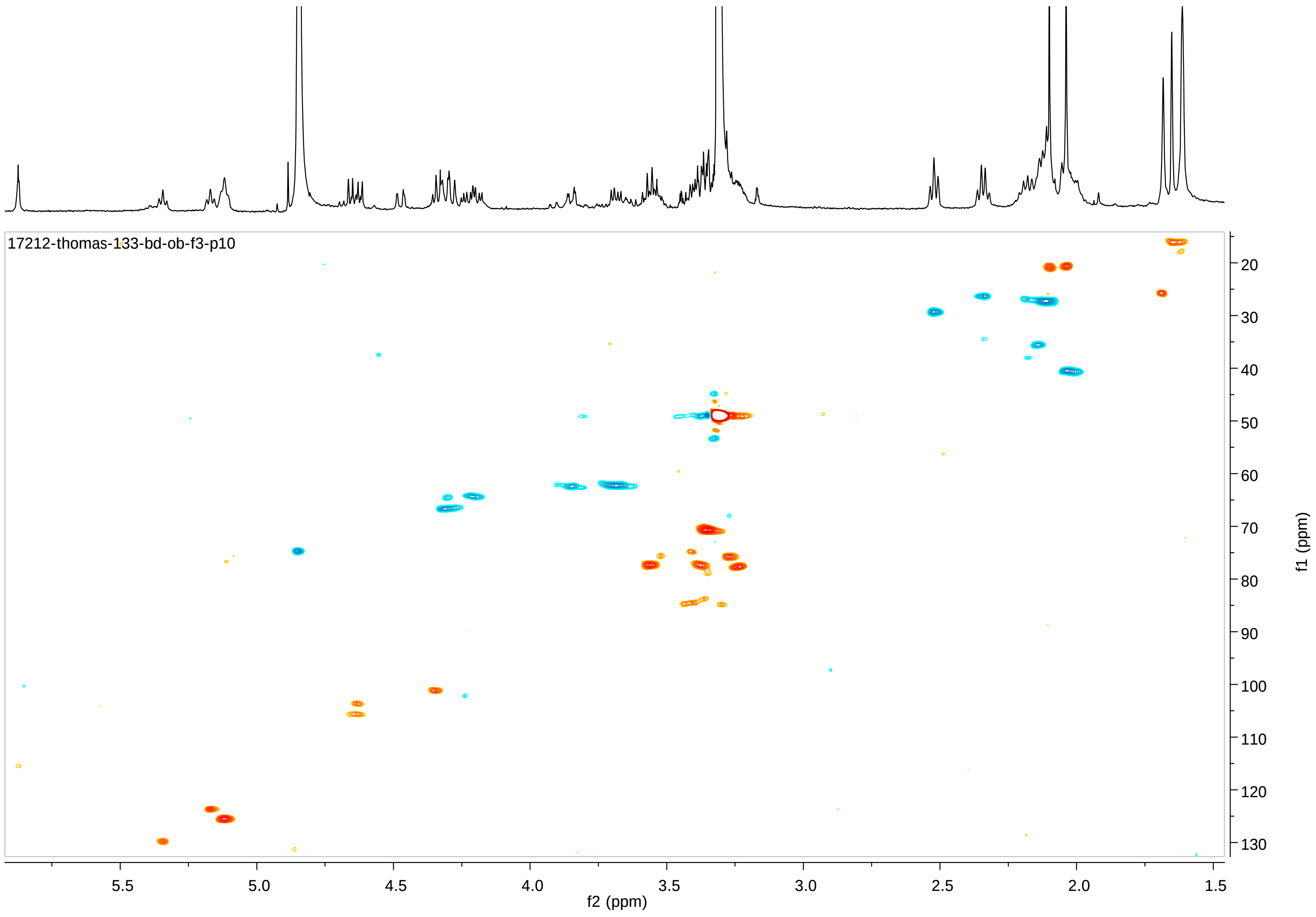

Figure S18. HRESIMS of **4**.

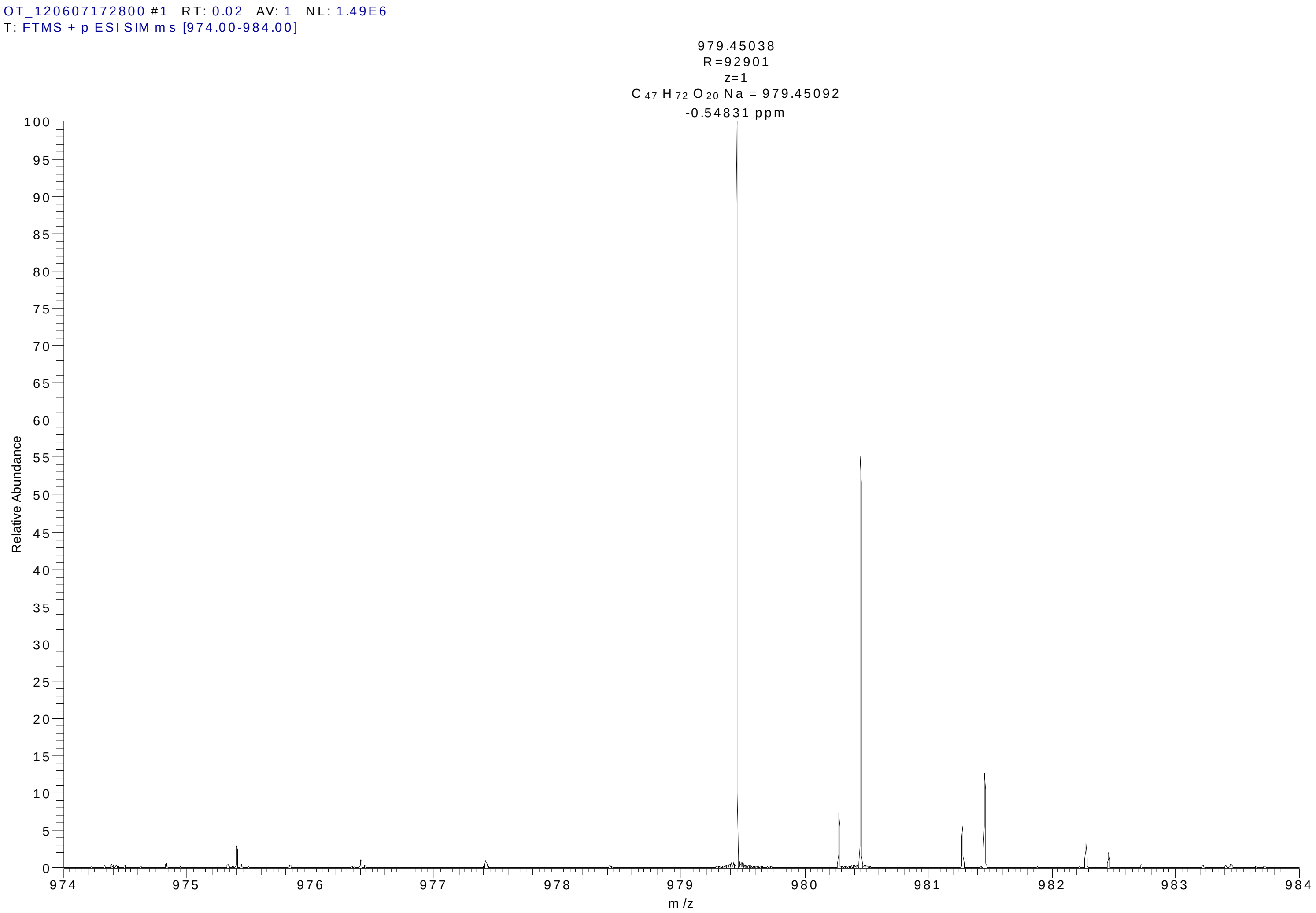

Supplement: Supplementary File 1 — Supplementary Information (PDF, 1587 KB) [file marinedrugs-11-01477-s001.pdf]
